# Endothelial ENaC as a repressor of oxidative stress and a guardian of lung capillary barrier function in bacterial and viral pneumonia

**DOI:** 10.3389/fphys.2025.1562626

**Published:** 2025-04-07

**Authors:** D. C. Eaton, M. J. Romero, M. A. Matthay, J. Hamacher, A. Advani, A. Wolf, M. Abu Mraheil, T. Chakraborty, D. W. Stepp, E. J. Belin de Chantemèle, A. Kutlar, F. Kraft, M. Zeitlinger, P. Kranke, S. Frank, Y. Su, A. D. Verin, D. J. R. Fulton, M. Ushio-Fukai, T. Fukai, R. Lucas

**Affiliations:** ^1^ Department of Medicine, Emory School of Medicine, Atlanta, GA, United States; ^2^ Vascular Biology Center, Augusta, GA, United States; ^3^ Department of Pharmacology and Toxicology, Augusta, GA, United States; ^4^ Cardiovascular Research Institute, University of California at San Francisco, San Francisco, CA, United States; ^5^ Pneumology, Clinic for General Internal Medicine, Lindenhofspital, Bern, Switzerland; ^6^ Lungen-und Atmungsstiftung, Bern, Switzerland; ^7^ Medical Clinic V-Pneumology, Allergology, Intensive Care Medicine, and Environmental Medicine, Faculty of Medicine, Saarland University, University Medical Centre of the Saarland, Homburg, Germany; ^8^ Department.of Medicine, Medical College of Georgia at Augusta University, Augusta, GA, United States; ^9^ Institute for Medical Microbiology, German Centre for Infection Giessen-Marburg-Langen Site, Faculty of Medicine, Justus-Liebig University, Giessen, Germany; ^10^ Medical University of Vienna, Department of Anaesthesia, Intensive Care Medicine and Pain Medicine, Clinical Division of General Anaesthesia and Intensive Care Medicine, Vienna, Austria; ^11^ Department of Clinical Pharmacology, Medical University of Vienna, Vienna, Austria; ^12^ Department of Anesthesiology, Critical Care, Emergency and Pain Medicine, University Hospital of Würzburg, Würzburg, Germany; ^13^ Department of Anaesthesiology, LMU University Hospital, LMU, Munich, Germany; ^14^ Research Service, Charlie Norwood Veterans Affairs Medical Center, Augusta, GA, United States

**Keywords:** NADPH oxidase, epithelial sodium channel, capillary endothelium, alveolar-capillary barrier function, pneumonia, TNF, TIP peptide

## Abstract

The endothelium represents a crucial regulator of vascular homeostasis. Since endothelial cells mainly rely on glycolysis rather than on oxidative phosphorylation for their ATP generation, this allows capillaries to transport the maximum amount of oxygen to oxygen-starved tissues, where it can be used for energy generation. However, the occasionally high levels of oxygen and of reactive oxygen species (ROS) in the blood vessels requires a balancing act between pro- and anti-oxidative mechanisms in the endothelium. When this balance is disturbed by excessive oxidative stress, as can occur in bacterial and viral pneumonia, endothelial barrier function can be compromised. This review will discuss some of the recently discovered barrier-protective mechanisms during bacterial and viral pneumonia, mediated through the reduction of oxidative stress in lung capillaries by the epithelial sodium channel (ENaC).

## Introduction

The increased levels of oxygen over time in the earth’s atmosphere provided the crucial fuel for the evolution of complex, multicellular organisms with high energy demands. Energy production through aerobic respiration comes with the risk of the generation of harmful incompletely reduced reactive oxygen species (ROS). The three primary species include the superoxide anion (O_2_^•−^), hydrogen peroxide (H_2_O_2_) and the hydroxyl radical (HO^•^). All of these are all oxygen-containing compounds with reactive properties. ROS represent very potent oxidants that, when present at high concentrations, can cause physiologic dysfunction within the cells (Hsia et al., 2013). In order to minimize ROS toxicity, humans have evolved the circulatory system with hemoglobin in erythrocytes to deliver oxygen efficiently to tissues, thereby preventing the accumulation of excessive oxygen in other areas of the body (Ortiz-Prado et al., 2019). Pulmonary venous and capillary endothelial cells are particularly challenged by oxidative stress, since they are at times exposed to O_2_-rich environments. To maximize O_2_ delivery to tissues and perivascular cells, these cells use the glycolytic pathway (Xiao et al., 2021), rather than mitochondrial respiration -classically used by most other eukaryotic cells-as the main ATP-generating pathway.

Optimal gas exchange between the alveoli and the lung capillaries requires tight barriers. The endothelial barrier, made up of adherens junctions and tight junctions, is essential for controlling the leakage from blood vessels into tissues and moreover represents the first line of defense against inflammatory or infectious insults in the vasculature (Dejana, 2004). Dysregulated endothelial permeability significantly contributes to morbidity and mortality. This is particularly relevant in microvascular endothelial beds, like in lung capillary endothelial cells in the acute respiratory distress syndrome (ARDS) (Price and Garcia, 2024; Wick et al., 2024; Tzotzos et al., 2020; Gonzales et al., 2015) (the definition of which was recently updated (Matthay et al., 2024) and in microvascular endothelial cells in the blood brain barrier as was shown in mouse models of Alzheimer disease (Hossain et al., 2024).

The endothelial cells associated with different parts of the vasculature appear to have somewhat different properties from one vascular bed to another. Lung microvascular endothelial cells (MVEC) are not characterized as well as the endothelial cells of larger vessels, but they are adapted to be highly permeable to promote gas exchange and movement of low molecular weight organics and ions without normally being permeable to serum proteins. One membrane protein, the epithelial sodium channel (ENaC), has been described in several endothelial beds, but until recently its presence was not demonstrated in MVEC. Using commercially available primary cultures of human lung microvascular endothelial cells (HL-MVEC; Lonza Biosciences), our group recently described functional endothelial ENaC using electrophysiological and pharmacological methods (Czikora et al., 2017; Romero et al., 2024). Studies with endothelial ENaC-α knockout mice indicated an important role of the endothelial ENaC channel for capillary barrier function in models of bacterial pneumonia (Romero et al., 2024). Although mechanisms to counteract oxygen toxicity, including ROS-neutralizing antioxidant enzymes like superoxide dismutase (SOD), glutathione peroxidase and catalase have been well studied, this short review will discuss recent discoveries regarding the interaction between the epithelial sodium channel (ENaC) and pro-oxidative mechanisms in capillary endothelial cells and the relevance thereof for barrier function during bacterial and viral pneumonia.

### Patho-mechanisms of impaired pulmonary endothelial barrier function in pneumococcal pneumonia and ARDS

As pointed out above, efficient gas exchange in the lungs requires a functional alveolar fluid clearance (AFC) capacity by type 1 and 2 alveolar epithelial cells (AT1/2), as well as the preservation of a tight alveolar-capillary barrier. This barrier not only facilitates diffusion of O_2_ and CO_2_ between the alveoli and the blood capillaries, but also controls movement of fluid, proteins and cells from the vascular compartment and interstitial space into the alveoli (Su et al., 2024). Adjacent endothelial cells express both adherens and tight junctions and these transmembrane adhesive proteins promote homophilic interactions that provide a pericellular zipper-like structure along the cell border. Cell adhesion at adherens junctions requires vascular endothelial cadherin (VE-cadherin), which is in turn linked to intracellular proteins like β-catenin, plakoglobin and p120. At tight junctions, adhesion is mediated by claudins, occludin and members of the junctional adhesion molecule (JAM) family (Dejana, 2004).

A common characteristic feature in autopsies of patients that succumbed to ARDS is the severely altered permeability of the alveolar-capillary barrier (Price and Garcia, 2024). The increase in permeability is a result of dynamic changes in cytoskeletal structure and adherens junction disorganization, such as the detachment of VE-cadherin from the actin cytoskeleton (Su et al., 2024; Gonzales et al., 2014). These cytoskeletal changes are due to myosin light chain phosphorylation and/or microtubule rearrangement. Either of these can be induced by pro-inflammatory factors, such as thrombin and TNF or by bacterial and viral compounds, such as LPS (Gonzales et al., 2014; Song et al., 2023), pneumolysin (Lucas et al., 2012a; Lucas et al., 2012b; Chen et al., 2014; Batori et al., 2022) or the S1 subunit of the SARS-CoV2 Spike protein, which contains the receptor binding domain for human ACE2 (Romero et al., 2023).

To improve gas exchange in patients with severe ARDS, high ventilator pressures are often required, but these can further aggravate barrier dysfunction and inflammation and as such induce ventilator-induced lung injury (VILI) (Johannes et al., 2014; Vieillard-Baron et al., 2016). Currently, barrier-strengthening Tie2-based strategies using the Tie2 agonist vasculotide are being evaluated in preclinical animal models combining bacterially-induced pneumonia with high ventilation pressures (Lask et al., 2022). Nonetheless, despite recent advances in the treatment of ARDS, i.e., low tidal volume ventilation (Amato et al., 1998; Brower et al., 2000), ventilation in a prone position (Gattinoni et al., 2001) and neuromuscular blockade (Huang et al., 2017), mortality remains high at about 40% (Wick et al., 2024). There is currently a complete lack of pharmacologic treatments for severe ARDS which is refractory to conventional therapy. Therefore the identification of novel therapeutic targets in ARDS associated with severe pneumonia remains an area of high priority.

A major complication in the development of pharmaceuticals to treat ARDS is the existence of hyper- and hypo-inflammatory patient phenotypes, each with distinct transcriptional and metagenomic features (Sinha et al., 2023; Neyton et al., 2024). A cohort of critically ill sepsis patients with hyperinflammatory ARDS had significantly increased levels of glycolytic metabolites such as lactate and pyruvate compared with patients with hypo-inflammatory ARDS, suggesting the involvement of excessive glycolysis in the pathology of hyper-inflammatory ARDS (Alipanah-Lechner et al., 2023). Moreover, following a transition from the hyper-to the hypo-inflammatory state survival of ARDS patients improved (van Amstel et al., 2024). A major challenge in developing novel pharmacological agents to treat ARDS is the recent observation of the divergent reactions to corticosteroid treatment in hyper-*versus* hypo-inflammatory phenotypes, reducing mortality in the former, but increasing it in the latter patient group (Sinha et al., 2021).

Major comorbidities that occur with ARDS are bacterial and viral pneumonia (Ha et al., 2024; Vaughn et al., 2024). One of the main etiological agents of mortality in children under 5 years of age worldwide and of community-acquired pneumonia (CAP) in the elderly is the facultative anaerobe, Gram-positive bacterium, *Streptococcus pneumoniae*. Mortality rates in individuals with CAP vary according to the treatment setting, with less than 1% in outpatient care, up to 18% in hospital wards and even reaching 47% in the intensive care unit (ICU) (Jain et al., 2015; Cavallazzi et al., 2020).

Pathological specimens from ARDS patients reveal diffuse alveolar damage. Moreover, animal studies of bacterial pneumonia-associated ARDS have demonstrated both alveolar epithelial and lung endothelial injury with accumulation of protein-rich fluid in the alveolar space (Zhang et al., 2024). The ability of pneumococci to promote lung disease in the human host depends not only on microbial virulence factors, such as the pore-forming toxin pneumolysin (Witzenrath et al., 2006) and H_2_O_2_ (Mraheil et al., 2021), but also on the age and on genetic and environmental factors. All of these affect the concerted ability of the immune system to clear bacteria on the one hand and the susceptibility to develop subsequent tissue damage on the other hand (Ochoa-Gondar et al., 2023).

### Regulation of vascular ROS production

Upon stimulation by inflammatory mediators such as TNF, otherwise quiescent endothelial cells become activated to produce significantly increased levels of NADPH oxidase-generated ROS (Gertzberg et al., 2004) with increased glycolysis mediated by 6-phosphofructo-2-kinase/fructose-2,6-biphosphatase (PFKFB3) (Cao et al., 2019). ROS can in turn combine with nitric oxide (NO) to generate barrier-disruptive peroxynitrite (Neumann et al., 2006). Reduction of endothelial nitric oxide synthase (eNOS)-mediated NO generation was shown to promote pulmonary microvessel leakage in eNOS^−/−^ mice (Predescu et al., 2005), suggesting a barrier-protective effect of eNOS-derived NO in the pulmonary microvasculature (Di Lorenzo et al., 2013). Increased ROS generation was shown to impair the expression and activity of eNOS (Neumann et al., 2004) and to increase the activity of arginase 1, an enzyme competing with eNOS for the common substrate arginine (Romero et al., 2008; Chandra et al., 2012). TNF appears to be critical for arginase 1 induction, since TNF^−/−^ mice had significantly reduced endothelial arginase activity following ischemia and reperfusion (Gao et al., 2007). All of these events involve increased endothelial oxidative stress and can disrupt the vascular integrity by reducing adherens and tight junction protein expression to eventually induce endothelial cell death. Moreover, ROS can increase expression of adhesion molecules in endothelium and as such foster transendothelial migration of circulating leukocytes (Wang and Doerschuk, 2000). Severely restricted blood flow, as can occur in TNF-induced multiple organ failure or in the lungs following lung transplantation, activates the mechano-sensing machinery in the endothelium that generates NADPH oxidase-mediated ROS to subsequently drive neutrophil influx (Noel et al., 2013).

Endothelial cells express several ROS-generating enzymes, including the NADPH oxidases 1, 2, 4 and 5 (NOX-1, -2, -4 and -5). Of these, NOX-2, in particular, has been shown to contribute to endothelial ROS generation in inflammatory conditions associated with infection and ischemia (Drummond and Sobey, 2014; Violi et al., 2015). Notably, the presence of soluble NOX2-derived peptide (sNOX2-dp), a marker of NOX2 activation, is increased in the circulation of patients with pneumococcal pneumonia (Violi et al., 2015; Violi et al., 2020) and in those with peripheral artery disease (Loffredo et al., 2013). However, NOX2-derived ROS generation can also confer beneficial activities in endothelium, since it stimulates angiogenesis in a mouse model of myocardial infarction (Youn et al., 2019).

**FIGURE 1:**
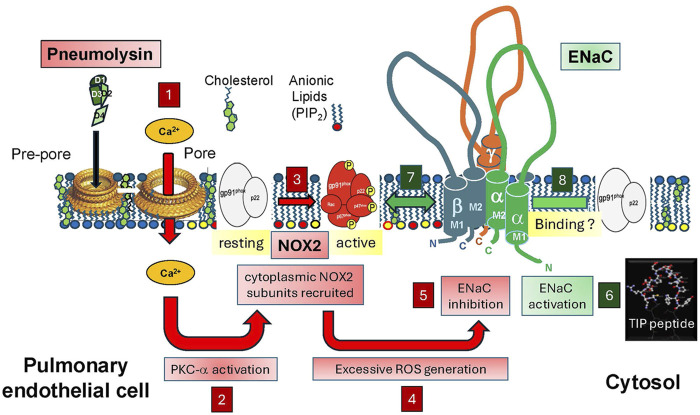
Upon autolysis, antibiotics treatment or upon induction by H_2_O_2_, *S. pneumoniae* (*Sp.*) releases the cholesterol-binding toxin pneumolysin, which, following pore formation, induces Ca^2+^ influx in infected cells (box 1). Increased intracellular Ca^2+^ activates PKC-α (box 2), which in turn phosphorylates NADPH oxidase 2 (NOX2) cytoplasmic subunits (box 3), thereby promoting their recruitment to the surface membrane, required for full activation of the enzyme. Increased NOX2 activity (box 4) promotes excessive generation of ROS in lung endothelial cells, which, together with the PKC-α activation, impairs ENaC activity (box 5). ENaC, activated by TIP peptide (box 6) can act as a repressor of NOX2 expression potentially upon binding to the gp91^phox^ subunit (box 7), thus inhibiting assembly of the NOX2 complex or it can inhibit PKC-α-mediated phosphorylation of the subunits and thus NOX2 activation (box 8). Based on our findings, we hypothesize that during pneumococcal pneumonia, direct activation of ENaC by the TIP peptide reduces pneumococci-induced endothelial NOX2 activation and as such improves capillary barrier function, without affecting bactericidal activity of neutrophils, which do not express ENaC.

Pneumococcal infection can cause extensive ROS generation in pulmonary microvascular endothelium (Li et al., 2018). The pore-forming toxin pneumolysin is the most important virulence factor produced by *S. pneumoniae* (Anderson and Feldman, 2017). It has been proposed as the main inducer of ROS generation in mammalian cells (Martner et al., 2008). Upon pore formation following the binding to cholesterol-containing membrane surfaces, pneumolysin induces a rapid influx of Ca^2+^, which can activate protein kinase C-α (Lucas et al., 2012a) and this in turn potently activates NOX2 (Schröder et al., 2017; Shafique et al., 2017). NOX2 activation requires the formation of a complex consisting of two surface membrane proteins: p22^phox^ and gp91^phox^ with the four cytosolic proteins p47^phox^, p67^phox^, p40^phox^ and the small GTPase Rac. The cytosolic subunits need to be phosphorylated by kinases like PKC in order to be recruited to the surface membrane (Schröder et al., 2017) (Figure 1). The role of NOX2 in pneumolysin-induced barrier dysfunction is also supported by the observation that a specific peptide inhibitor of NOX2 -gp91dstat-significantly blunts hyperpermeability induced by the toxin in human lung microvascular endothelial cell (HL-MVEC) monolayers, as measured by electrical cell substrate impedance sensing (Romero et al., 2024).

Another source of increased endothelial ROS generation are defective mitochondria. Apart from activating NOX2, pneumolysin also induces mitochondrial dysfunction which increases mitochondrial ROS generation in human lung MVEC (Romero et al., 2024; Shafique et al., 2017). It should be noted that a cross talk between mitochondrial ROS and NOX-derived ROS was recently suggested and that this intracellular communication could represent a ROS amplification mechanism in distinct subcellular compartments, relevant for activation of redox signaling (Shafique et al., 2017; Fukai and Ushio-Fukai, 2020).

### H_2_O_2_ as a virulence factor of pneumococci

Apart from superoxide generated by host cells upon pneumococcal-induced NOX2 activation, *S*. *pneumoniae*, which lacks catalase and common regulators of peroxide stress resistance, can by itself generate and release millimolar levels of H_2_O_2_ as a virulence factor (Mraheil et al., 2021). These H_2_O_2_ levels are high enough to kill or inhibit the growth of other common inhabitants of the respiratory tract, such as *Haemophilus influenzae* and *Staphylococcus aureus*, presumably as an evolutionary strategy to promote *S. pneumoniae* colonization (Regev-Yochay et al., 2006).

The main source of H_2_O_2_ generation in pneumococci is the enzyme pyruvate oxidase, which catalyzes the conversion of pyruvate to the phosphoryl donor, acetyl phosphate (Ac-P), while releasing CO_2_ and H_2_O_2_ as by-products (Mraheil et al., 2021). Another enzyme, lactate oxidase (LctO), positively impacts pyruvate flux through pyruvate oxidase, since it converts lactate to pyruvate. Importantly, actions of H_2_O_2_ overlap and complement those of pneumolysin in modulating the host immune responses and promoting organ injury (Pericone et al., 2000). As such, the release of pneumolysin, which normally occurs in the lungs by autolysis or after antibiotic-mediated lysis is defective in pneumococcal mutants that lack the pyruvate oxidase gene, whereas it is restored upon complementation of the enzyme. Since catalase supplementation, but not exogenous H_2_O_2_, prevents the release of pneumolysin in some pneumococcal strains, indicating the involvement of intracellular, rather than secreted H_2_O_2_ in this process (Bazant et al., 2023). Infection of H441 cells with wild type pneumococci was demonstrated to alter the kinome of the cells. This occurs at least partially through H_2_O_2_-mediated downregulation of Protein kinase B (Akt1) and activation of lymphocyte-specific tyrosine protein kinase (Lck) via H_2_O_2_-mediated phosphorylation (Bazant et al., 2024).

### Dual role of NOX2 in pneumococcal pneumonia

While there has been a focus on the pathogens causing pneumonia in recent years*,* there is an urgent need for research from the perspective of the host, especially in order to develop strategies to protect lung barrier function and alveolar fluid clearance. The problem is that ROS, such as H_2_O_2_ and superoxide play an important but highly complex role in pneumococcal pneumonia-associated ARDS. On the one hand, ROS generation is a conserved strategy of host phagocytic cells -primarily neutrophils, monocytes and macrophages-to facilitate clearance of bacteria at the infection site. Bacteria can be engulfed and enclosed in phagosomes, into which superoxide is released by activated NOX2. The resulting superoxide O_2_^−^ then dismutates to H_2_O_2_ through the action of superoxide dismutase (SOD) in macrophages. H_2_O_2_ can then further be converted by myeloperoxidase (MPO) in neutrophils to hypochlorous acid, a highly microbicidal species (Winterbourn et al., 2016).

On the other hand, ROS generated in alveolar epithelial and capillary endothelial cells can impair alveolar liquid clearance mechanisms and contribute to dysfunction of the capillary endothelial barrier, respectively. In view of the dual role of NOX2 in pneumonia, strategies that globally reduce NOX2 activity bear the risk of increasing susceptibility to infections (Diebold et al., 2015). Novel therapies that can reduce deleterious oxidative stress in the alveolar epithelium and capillary endothelium, without significantly affecting anti-bacterial ROS generation in phagocytes seem therapeutically promising in pneumonia and ARDS.

### Effects of ROS on sodium transport in alveolar epithelial cells

Of all organs in the human body, the lungs are exposed to the highest levels of free O_2_, mainly originating from inspired air, with concomitant increased ROS production. Shortly after birth, the newborn lung must adapt from an embryonic fluid-secreting tissue to a fluid-absorbing organ, in order to allow for breathing normal ambient air. In particular, AT1/2 are exposed to the relatively high levels of free O_2_ in inspired air, which can lead to the generation of additional ROS. As such, physiological concentrations of H_2_O_2_ in the alveolar space in man are in the 1–10 μM range (Corradi et al., 2008).

Most ion transporters evolved from simpler channel-forming peptides following intragenic duplication events into more complex multi-domain transporters with diverse functions. The epithelial sodium channel (ENaC), consisting in its native configuration of three subunits: α, β and γ, is an example of such a multifunctional ion channel, since, apart from controlling blood pressure, it also modulates alveolar fluid clearance (AFC), by regulating vectorial Na^+^ transport in AT1/2 cells to control alveolar fluid balance and thereby assure normal gas exchange in the lungs (Matalon and O'Brodovich, 1999; Hummler et al., 1997).

Perinatal exposure of resident AT1/2 cells to O_2_-derived ROS was proposed to deliver a signal for initiating the uptake of Na^+^ by ENaC. This idea is supported by the observation that an increase in oxygen tension of up to 100 mmHg can augment Na^+^ transport within 6 h in fetal distal lung epithelial (FDLE) cells (Baines et al., 2001).

The overall Na^+^ transport capacity of ENaC is determined by the product of the channel’s surface density (*N*) and the mean open probability of each individual channel (*Po*); thus, by the product *NPo*. Na^+^ is then secreted in the interstitial space by the basolateral Na^+^-K^+^-ATPase. This vectorial Na^+^ transport is necessary to promote fluid clearance (AFC) from the newborn and the adult lung. The crucial role of ENaC in this process is underscored by the observation that mice lacking the pore-forming α subunit of ENaC die of alveolar flooding at birth (Hummler et al., 1996). Impairment of AFC capacity correlates with morbidity and mortality in patients with the acute respiratory distress syndrome (ARDS) (Ware and Matthay, 2001).

The increase in sodium transport postnatally is at least partially due to both an increase in ENaC open probability and in surface protein expression stimulated by ambient oxygen production of low levels of ROS. H_2_O_2_ activates PI3-kinases, which in turn generate the anionic phospholipids, such as phosphatidylinositol-3,4,5-trisphosphate (PIP_3_), which increases ENaC open probability and AFC (Kooijman et al., 2011; Pochynyuk et al., 2006; Yue et al., 2002). Long-term exposure to elevated O_2_ tension or moderate levels of ROS can increase activity of ENaC subunit gene promoters, since these contain redox-sensitive NF-κB and AP-1 response elements and can moreover promote total ENaC protein expression (Yue et al., 2002; Rafii et al., 1998; Thome et al., 2003). Apart from a direct sensitivity of ENaC in AT1/2 cells to O_2_, ROS generated by increased O_2_ tension, such as superoxide anion (O_2_^−^) also has the capacity to regulate ENaC as shown by the observation that in cellular models of alveolar fluid uptake addition of the cell-permeable O_2_^−^ scavenger TEMPOL significantly decreases ENaC activity (Bremner et al., 2002).

### Endothelial ENaC represses oxidative stress in capillaries during bacterial and viral pneumonia

Like AT1/2 cells, also human lung microvascular endothelial cells (HL-MVEC) express all three ENaC subunits (albeit at a significantly lower protein expression levels) (Yu et al., 2007). HL-MVEC generate electrophysiologically-identified cation channels with a conductance of 5 picoSiemens, and a current voltage relationship characteristic of functional ENaC channels (Romero et al., 2024). However, the role of ENaC and even the composition of sodium transporting channels in endothelium is not completely understood.

As discussed above, a low basal level of NOX2-mediated ROS generation is necessary for proper ENaC activity in AT1/2 cells (Takemura et al., 2010). HL-MVEC treated with specific ENaC-α siRNA or mouse lung ECs isolated from tamoxifen-inducible endothelial ENaC-α KO mice (produced by cross-breeding conditional *Scnna* ^lox/lox^ mice (Hummler et al., 2002) with tamoxifen-inducible VE-cadherin–CRE/ert2 driver mice (Wang et al., 2010) have a significantly higher protein expression level of the gp91^phox^ NOX2 subunit than human lung MVEC transfected with scrambled siRNA or mouse lung ECs from control CRE driver mice or. Mouse lung endothelial cells from endothelial ENaC-α KO mice generated much higher levels of superoxide than cells from control animals upon stimulation with PMA (Romero et al., 2024). These findings indicate that ENaC-α inhibits NOX2 expression in lung endothelial cells from both mice and humans. Although further studies are required, recent findings from co-immunoprecipitation experiments in mouse lungs substantiate earlier findings of a direct binding between gp91^phox^ and ENaC-α (Romero et al., 2024). Such an interaction might also occur in lung endothelial cells and could potentially interfere with NOX subunit assembly and successful NOX2 complex formation. An alternative mechanism by which activated endothelial ENaC could blunt NOX2 activity is through the inhibition of enzymes phosphorylating cytoplasmic subunits, necessary for their recruitment to the surface membrane and for complex assembly of the functional enzyme (Schröder et al., 2017). One such enzyme which is able to activate NOX2 is protein kinase C-α (PKC-α), the activity of which is increased by pneumolysin (Lucas et al., 2012b).

While these observations suggest that ENaC-α is necessary for normal endothelial cell function and serves to keep NOX2 activity at bay, the results do not necessarily show that hetero-multimeric ENaC is required. Classical ENaC in epithelial tissues consist of α, β, and γ subunits, but in renal endothelial cells there is some evidence that ENaC-α may form alternative combinations without one or more of the regulatory β or γ subunits (Mutchler et al., 2021; Trac et al., 2017). These non-classical channels can therefore also potentially alter endothelial function. To complicate the issue further, in human endothelial cells there is a fourth ENaC subunit, δ, which can substitute for ENaC-α to form sodium permeable ion channels (Ji et al., 2012). Whether any of these alternative constructs can alter endothelial function as native ENaC does, is however unclear to date. However, this does mean that results implicating ENaC in endothelial cells must be interpreted carefully especially when pharmacological agents are used to observe cellular responses to specific molecules.

In order to further investigate the effect of specific ENaC activation on endothelial NOX2 activity, we have used the TNF-derived TIP peptide (a.k.a. AP301, Solnatide), generated by our group (Lucas et al., 1994). This 17 residue cyclic synthetic peptide with the sequence CGQRETPEGAEAKPWYC, mimics the lectin-like domain of human TNF (Lucas et al., 1994; Elia et al., 2003) and increases ENaC activity upon binding to a helical structure in the C-terminal domain of the α subunit (Czikora et al., 2014; Lucas et al., 2016; Martin-Malpartida et al., 2022). Another direct peptide ENaC activator is compound S3969, which interacts with a specific binding pocket in the channel’s β-subunit (Lu et al., 2008; Sure et al., 2024). TIP peptide can increase the open probability as well as the surface expression of ENaC in human lung MVEC and in alveolar epithelial cells, even following treatment of the cells with pneumolysin (Romero et al., 2024; Lucas et al., 2016). Pneumolysin rapidly reduces ENaC open probability and moreover reduces surface expression of the crucial α subunit, possibly by decreasing lysine residue acetylation, which in turn increases its ubiquitination and degradation (Romero et al., 2024; Butler et al., 2015). Moreover, pneumolysin-induced mitochondrial ROS generation -which exerts cross-talk with NOX2-derived ROS- is blunted by TIP peptide in HL-MVEC (Romero et al., 2024).

It should be stressed that the TIP peptide is a specific and direct activator of ENaC, unlike other used indirect activators, such as aldosterone, the latter of which can also exert ENaC-independent effects, which may chronically affect vascular function through the paracrine release of histamine (Schjerning et al., 2013). The barrier-disruptive actions of pneumolysin on HL-MVEC barrier function involve NOX2 activation, since we showed that the specific inhibitor gp91dstat was protective (Romero et al., 2024). We found a similar protective activity towards pneumolysin-induced hyperpermeability in HL-MVEC monolayers with the TIP peptide, as such supporting our published hypothesis that activation of endothelial ENaC represses NOX2 expression and activity and at least partially as such strengthens capillary barriers in pneumococcal pneumonia (Czikora et al., 2017; Romero et al., 2024).

Although a barrier strengthening effect of ENaC-α in LPS-treated capillary endothelium was also reported in other studies (Sternak et al., 2018), other groups, often using aldosterone, have reported deleterious vascular stiffening actions of ENaC in studies ln large vessel endothelial cells (Jia et al., 2023; Zhang et al., 2022). TIP peptide inhibits PMA-induced phosphorylation of the cytoplasmic NOX2 subunit p47^phox^ and it partially blunts PMA-induced ROS generation in COSp22^phox^ cells, which express ENaC-α and NOX2, but not other NOX enzymes. By contrast, TIP peptide does not blunt PMA-induced ROS generation in primary bone marrow-derived mouse PMNs, which do not express ENaC-α (Romero et al., 2024). Taken together, these data demonstrate that ENaC, and especially its α subunit represents a novel repressor of PMA-induced NOX2 activity in microvascular endothelium, but not in PMNs, especially when activated by the TIP peptide (Figure 1).

In a mouse model of pneumococcal pneumonia-induced lung injury, a moderate dose of pneumolysin (1.5 μg/kg) or a low inoculum of D39 pneumococci (2 × 10^6^ CFU) induces significantly higher capillary leak (measured as Evans Blue permeability from the vasculature into the alveoli) in tamoxifen-inducible endothelial ENaC-α KO mice as compared to control CRE driver mice. However, co-instillation of 2.5 mg/kg of TIP peptide significantly blunts capillary leak induced by a high inoculum of *S. pneumoniae* (10^7^ CFU), without significantly affecting bacterial load in lung homogenates (Romero et al., 2024). These results demonstrate an important barrier-protective function for endothelial ENaC-α in murine pneumococcal pneumonia (Figure 1).

TIP peptide also blunts ROS generation and barrier dysfunction in HL-MVEC induced by the S1 subunit of the SARS-CoV2 spike protein (Romero et al., 2023), as such indicating that endothelial ENaC can also suppress endothelial oxidative stress in COVID-19 and possibly in long COVID, where the spike protein, which can be detected months later even after the virus is no longer present, was proposed to be involved in the pathology (Iba et al., 2024).

### Clinical trials evaluating the TIP peptide in patients with lung transplantation and ARDS

In a phase 1 clinical trial in healthy volunteers, inhalation of the ENaC-activator TIP peptide (a.k.a AP301, Solnatide) caused no noticeable side effects (Schwameis et al., 2014). A phase 2a double-blind trial was done in lung transplantation patients, the design of which was based on a preclinical study in rats with lung iso-transplantation showing potent anti-oxidative actions and lung function-promoting of the TIP peptide (Hamacher et al., 2010). Ischemia-reperfusion following lung transplantation was suggested to induce endothelial oxidative stress involving NOX2 activation following mechano-signaling (Chatterjee et al., 2015). The trial demonstrated a significant reduction in days on the ventilator in patients inhaling the peptide twice daily over 7 days, as compared to placebo (Aigner et al., 2017; Ware, 2017). A similar treatment pattern with the test compound in patients with ARDS revealed a significant reduction in extravascular lung water in ARDS patients with a sequential organ failure assessment (SOFA) score >11 (Krenn et al., 2017). Currently, a multi-center double-blind dose ascending phase 2b clinical trial is ongoing in a group of non-COVID and COVID ARDS patients receiving the TIP peptide (a.k.a. Solnatide) during invasive ventilation, organized by the Vienna-based Biotech company Apeptico. Results from this trial should provide further information regarding the peptide’s therapeutic potential (Schmid et al., 2021; Schmid et al., 2022).

## Discussion

Our data from mouse studies and cell studies with human lung MVEC have demonstrated that the epithelial sodium channel, and especially its α subunit, apart from playing a crucial role in vectorial Na^+^ transport and fluid clearance in the alveolar space, also represents a novel repressor of oxidative stress and a guardian of lung capillary barrier function during experimental pneumococcal and SARS-CoV2-induced pneumonia. These lung-protective mechanisms can be activated by a direct ENaC activator, the TNF-derived TIP peptide, in models of pneumococcal and SARS-CoV2-induced pneumonia. As a limitation of our studies, we should note that although we demonstrated ENaC expression and activity in human lung microvascular endothelial cells, expression of the channel in mouse lung capillaries has not been visualized so far. This may be complicated by low ENaC expression levels as well as by the existence of different capillary sub-phenotypes. Since capillary endothelial cells make up the majority of lung endothelial cells and since mice lacking endothelial ENaC alpha display significantly increased capillary leak as compared to control animals in pneumococcal pneumonia, this points to an important role of the channel in lung capillaries, although the involvement of other microvascular beds cannot be excluded. The blood-perfused isolated human lung (Ross et al., 2019) may represent a more translational model system for further evaluation of the observed effects of endothelial ENaC in pneumonia and ARDS in man.

Although other groups have demonstrated a protective effect of ENaC-α on capillary barrier function in the presence of LPS and on vasodilation capacity of large vessels (Sternak et al., 2018; Tarjus et al., 2017), more research is required to explain the reported deleterious role of endothelial ENaC in vascular stiffening (Jia et al., 2023; Zhang et al., 2022; Kusche-Vihrog et al., 2014). Whether these divergent observations regarding the role of ENaC in endothelial function can be explained by differences between capillary and large vessel endothelial cells, the use of direct (e.g., TIP peptide, S3969) *versus* indirect (e.g., aldosterone) activators of ENaC, by the existence of alternative ENaC-like channels in endothelium or by differences in the disease models used remains to be investigated.

Although increases in the Renin-Angiotensin System (RAS) can lead to increased absorption of Na^+^ through ENaC in the distal nephron, the TIP peptide -a direct ENaC activator-did not increase blood pressure in a mouse model of glomerulonephritis (Madaio et al., 2019). One possible explanation for this is that the TIP peptide, which mimics the lectin-like domain of TNF, can bind to uromodulin in the loops of Henle (Hession et al., 1987) before reaching the distal nephron (Madaio et al., 2019).

Phase 2 clinical trials with inhaled TIP peptide in lung transplantation (Hamacher et al., 2010) and ARDS patients (Krenn et al., 2017) have shown encouraging outcomes on lung function, indicating that activation of ENaC may be a promising approach in these pathologies. Negative effects of the treatment on vascular function or blood pressure have not been observed in these trials, although this may be linked to the rather modest number of subjects tested so far or to the limited leakage of the test compound into the blood circulation.
